# Notes and News

**Published:** 1889-11-16

**Authors:** 


### Notes and News.

Collected and Communicated.

We shall be glad to receive as soon as possible the appeals of hospitals, nursing, and philanthropic institutions for insertion in our Special New Year's Number. All such appeals should distinctly state the specific needs, the amount of money or gifts required, also what the work, the expenditure, and the income by subscriptions and donations of the institution has been during the last year.

And now we are talking of Christmas we would ask every lady reader of our paper to make at least one garment or one present for a hospital patient for that day. *Truth* has covered the ground so far as the children are concerned, but why should the adults be forgotten? Perhaps this request comes rather late for this year; still, we hope those who can help will do so, and we will publish the names of those institutions most in need of such helpful charity to brighten the Christmas season. Those who have no time for such work can at least send a little money to the Editor to be expended upon useful articles at his discretion.

Dr. Austin Meldon delivered the inaugural address at the opening of the session of Jervis-street Hospital, Dublin. He described Pasteur's method of innoculating dogs to prevent rabies. He said there was a natural tendency to resist the development of a microbe, and when the nervous system is strong successfully, but when the nervous system is weakened the microbe easily conquers. This theory is strengthened by the fact that if the poison of the charbon symptomatique is injected into a healthy rabbit no evil follows, but if the part injected is bruised or the animal weakened, then the disease is developed. Again, with regard to hydrophobia, it is well known that a large number of those who die of the disease are of nervous temperament. Epileptics seldom escape when bitten.

Derbyshire has been commemorating the eightieth anniversary of its General Infirmary. The president of the year (H. J. Wood, Esq., of Breadsall) attended Divine service at All Saints' Church, accompanied by the magistrates, members of the Corporation, and governors of the infirmary. The sermon was preached by the Right Rev. the Lord Bishop of Shrewsbury. The annual meeting was afterwards held in the board-room of the infirmary, when the President took the chair, and there was a large attendance of governors and subscribers. The cost of the patients has not varied materially from former years, each in-patient this year costing £4 9s. 10d., and each out-patient 3s. 7d.; the average cost during the past three years was £4 8s. per in-patient, and 4s. 1d. per out-patient. The total expenditure under the various heads was £6,699 7s. 11d., and the receipts £6,677 6s. 7d., or £22 1s. 4d. less than the expenditure of the year.

Northallerton Cottage Hospital issues its twelfth report, which is in every way satisfactory. The committee think of starting a district nurse, or else taking a probationer. Paying probationers have answered so well in cottage hospitals that we recommend them heartily to the committee; they do good work as a rule, and are an inexpensive luxury.

The quarterly paper of the Edinburgh Medical Missionary Society contains the record of an immense amount of work. From France, India, China, and Kashmir come letters from those who are working for the mission. It is suggested that in 1891 the jubilee of female medical missions should be celebrated in the form of a Medical Missionary Training Institution for Ladies.

Sir William Willis has written a special appeal for help for the Female Mission to the Fallen. During the past year 909 women have received the aid of the mission, of whom 227 have been placed in homes, 78 restored to their friends, 201 provided with situations, and 10 assisted to emigrate or marry. Besides these 909, several old cases have been again assisted. The average cost of each case aided is about £5.

The annual meeting of Doncaster Infirmary was held lately, the Mayor presiding. The number of in-patients has been 169, as against 200 last year, while the dispensary patients have numbered 2,189, as compared with 2,361. The total income from all sources has been £1,856 13s. 7d., while the expenditure has been £1,466 2s. 2d., so the committee have been able to invest the donations and sundry receipts, a result, they think, very satisfactory.

We are all ready enough to help when the helping does not cost too much. Therefore, when it is known that the modest hope is entertained by the heads of the Provident Dispensary at Boscombe, Bournemouth, of being able to provide a Christmas Tree festivity for certain little folk—members of that admirable society—who have never, many of them, seen such a sight, it is not too much to expect that kindly hearts and skilful fingers will be stirred to aid in carrying out the scheme. Parcels of toys, trifling gifts, or knitted articles will be gladly received by the Matron, Boscombe Cottage Hospital, Bournemouth.

The Hospital Saturday Committee of Coventry and Warwickshire Hospital have held their general meeting. During the year they have collected £1,026 16s., the expenses having been £35 1s. 9d., leaving the net sum to be handed over £991 14s. 1½d. The list of subscribers was read, the four largest being: Rudge and Co., £120; Cheylesmore Company, £96; Singer and Co., £90; and Hillman, Herbert, and Cooper, £81. The Chairman said at the time £1,000 was mentioned as a sum the working-men hoped to reach, it was looked upon as a dream, but the dream had been realised that night, which would be a memorable one.

While at Oxford the other day we heard an extraordinary story of the effects of cocaine used as an injection. A professor had the cocaine injected in his jaw, and immediately became unconscious; artificial respiration was resorted to, and after six hours consciousness returned. The professor says that though apparently lifeless he heard all that was going on around him; this is one more lesson to doctors and nurses to be very careful what they say in the presence of one they believe unconscious. The poor professor ever since he recovered has been haunted by the remembrance of a case of drowning, where he tried artificial respiration for two hours, and then gave in; had his doctors given in at the end of two hours, where would the professor be now?

The following vacancies are announced this week, the amount of the salary in each case being given after the name of the institution:

**Resident Medical Officers.**

Pendlebury Hospital. Junior Medical Officer—£80, board, &c.
North-Eastern Hospital for Children. Junior House Surgeon—£30.
London Temperance Hospital. House Surgeon.
Holloway Dispensary. Medical Officer—£150, rooms, &c.
Warneford Hospital. House Surgeon—£100, board, &c.
Parish of Eday, Orkney. Medical Officer—£70.
Sussex County Hospital. House Surgeon—£110, board, &c.

**Non-Resident Medical Officers.**

Woodstock Union. Workhouse and District Medical Officer—£75.
Owens College, Manchester. Senior Demonstrator in Physiology—£150.
London Hospital, Whitechapel. Surgical Registrar—£100.
Great Northern Central Hospital. Ophthalmic Surgeon.

Lady Meux has laid the foundation-stone of a cottage hospital for Cheshunt. The friendly societies and fire brigade attended the ceremony. Lady Meux said: "I declare this stone to be well and truly laid; and I endow this cottage hospital with £500." Lady Meux had already been a contributor of £300 to the building fund, besides inducing others to give, and loud cheers followed this announcement of unexpected generosity. The Vicar of Cheshunt read the prayers.

The sixth annual report of Morley House Convalescent Home for Working Men is chiefly interesting for the full particulars it contains. For instance, there is a diet table which might be useful to other homes, only we should like to have seen more varied breakfasts or suppers. Still the home has a debt of £600 yet to be cleared, so strict economy must be the rule. The home does good work, and doubtless some charitable person will soon see fit to rid it of its weight of debt.

The Rev. H. Tristram Valentine has been appointed to the vicarage of St. Paul's, Walden, and the post of chaplain to the London Hospital will shortly be vacant. Let us hope that some clergyman of broad and kindly principles and plenty of tact will be appointed to this difficult post. It is the glory of the London Hospital that its doors are open to all sects and creeds. Jew and Gentile, Catholic and Dissenter, all meet there on common ground, and to stir up strife amongst them is surely no Christian proceeding.

The extension of Waterford Union Hospital is now completed, and the Hon. Dudley Fortescue and the committee have formally inspected the building and pronounced it satisfactory. The extension is two storeys high, containing four large wards, each capable of accommodating about thirteen patients. Over fifty beds are thus provided, the addition being much required in consequence of the increase within recent years of the demands on the Union Hospital's resources. The building is very solidly erected in stone. The ventilation has been specially attended to, and all modern conveniences introduced.

Dr. Kendal Franks delivered the opening address at the Adelaide Hospital, Dublin, on November 4, taking as his chief subject the use of antiseptics. After quoting the enormous decrease in the loss of lives in war at present, as compared with the Crimea, Dr. Franks proceeded: In this hospital antiseptic surgery has flourished and has borne much fruit. Blood-poisoning and erysipelas have practically been abolished, and by its means numerous lives have been saved. The death rate after operations has been reduced to less than 3½ per cent., a point which would have been incredible a generation ago.

Birmingham Hospital Sunday Fund has exceeded £4,000, but not yet reached £5,000. All churches and chapels have worked for the cause. The Society of Friends gave £130, the Moseley Baptist Church, £30; but the largest sums were received from the Church of England services.

Coventry is also going in for a very perfect workhouse infirmary, though it is not yet nearly completed. There is an ingenious plan for flushing the drains. Outside the building, at each corner, near the bathrooms, there is a flushing chamber, in which the water from the baths accumulates, until 300 gallons has collected. Then, by means of an automatic syphon, it is allowed to rush into the drains, effectually flushing them.

The Society of Friends is great in medical missionary work, but these quiet folk are so retiring that their good deeds in India and elsewhere are little known. At Philippopolis a hospital has been opened which has just concluded its first year's work. Seventy-five in-patients were treated at a cost of £250. Dr. Almina Forquhar is a lady Friend who works at the medical mission at Beyrout. At Brumana a hospital has been established for some time, and a dispensary is now being built. It is probable that six more members of the Society of Friends will go out as missionaries to India and Madagascar before the close of the present year.

Just those things which we wish to prove infectious often do not do so. The Mayor of Carnarvon lately gave £500 towards a hospital, and the only other person who caught this attack of generosity was the chairman of the Sanitary Committee, who gave £100. There has been patient waiting, but the attack does not spread, so now it is necessary to consider whether a small hospital for the town only can be built, and the outlying districts be left in their present state. It is a pity in building a hospital that it should be too small for all who seek its aid, but if generosity won't spread in such a hard-hearted neighbourhood, this is what must be done.

Dr. Smyly has been elected to the mastership of the Rotunda Hospital, an election which was, in fact, a foregone conclusion. He is an examiner in midwifery at the College of Physicians, and has been attached as obstetric physician to the City of Dublin Hospital, while his contributions to the branch of the profession he has selected have been of a very high order. The Catholic party had persuaded Dr. Byrne and Dr. Madden to stand, and doubtless the election of either of these two gentlemen would have quieted the rather riotous feelings of many Dublinites. It is a great pity when the question of different creeds interferes with the one great duty of charity.

The sub-committee appointed to inspect other hospitals, with a view to a new infirmary for Halifax, have drawn up their report after visiting some fifteen of our best hospitals. Their recommendations are: 1. That the wards for patients of the new infirmary be built in pavilions, and not exceeding one storey in height, in addition to the requisite basement—the administrative portions of the buildings not being restricted in this respect. 2. That the required bedroom and sitting-room accommodation for the nurses be provided in a building detached from the infirmary proper. 3. That in proceeding to obtain plans for the new infirmary, the same principle be adopted which has been carried out in the recently erected Bath Hospital at Harrogate, and at the Great Northern Central Hospital, London—viz., the appointment of an architect of established reputation, who shall act as assessor, and shall assist the committee in drawing up the particulars and instructions to architects, and advise the committee as to the relative merits of the designs submitted to them.

